# Graph analysis of the anatomical network organization of the hippocampal formation and parahippocampal region in the rat

**DOI:** 10.1007/s00429-015-0992-0

**Published:** 2015-01-25

**Authors:** F. Z. M. Binicewicz, N. M. van Strien, W. J. Wadman, M. P. van den Heuvel, N. L. M. Cappaert

**Affiliations:** 1 Swammerdam Institute for Life Science, Center for Neuroscience, University of Amsterdam, Science Park 904, Room C3.266, 1098 XH Amsterdam, The Netherlands; 2 Kavli Institute for Systems Neuroscience and Centre for Neural Computation, Norwegian University of Science and Technology, Trondheim, Norway; 3 Department of Psychiatry, University Medical Center Utrecht, Utrecht, The Netherlands; 4 Brain Center Rudolf Magnus, University Medical Center Utrecht, Utrecht, The Netherlands

**Keywords:** Graph analysis, Hippocampus, Neural network, Parahippocampal region, Rat

## Abstract

Graph theory was used to analyze the anatomical network of the rat hippocampal formation and the parahippocampal region (van Strien et al., Nat Rev Neurosci 10(4):272–282, 2009). For this analysis, the full network was decomposed along the three anatomical axes, resulting in three networks that describe the connectivity within the rostrocaudal, dorsoventral and laminar dimensions. The rostrocaudal network had a connection density of 12 % and a path length of 2.4. The dorsoventral network had a high cluster coefficient (0.53), a relatively high path length (1.62) and a rich club was identified. The modularity analysis revealed three modules in the dorsoventral network. The laminar network contained most information. The laminar dimension revealed a network with high clustering coefficient (0.47), a relatively high path length (2.11) and four significantly increased characteristic network building blocks (structural motifs). Thirteen rich club nodes were identified, almost all of them situated in the parahippocampal region. Six connector hubs were detected and all of them were located in the entorhinal cortex. Three large modules were revealed, indicating a close relationship between the perirhinal and postrhinal cortex as well as between the lateral and medial entorhinal cortex. These results confirmed the central position of the entorhinal cortex in the (para)hippocampal network and this possibly explains why pathology in this region has such profound impact on cognitive function, as seen in several brain diseases. The results also have implications for the idea of strict separation of the “spatial” and the “non-spatial” information stream into the hippocampus. This two-stream memory model suggests that the information influx from, respectively, the postrhinal–medial entorhinal cortex and the perirhinal–lateral entorhinal cortex is separate, but the current analysis shows that this apparent separation is not determined by anatomical constraints.

**Electronic supplementary material:**

The online version of this article (doi:10.1007/s00429-015-0992-0) contains supplementary material, which is available to authorized users.

## Introduction

The anatomical tract-tracing studies performed in the last century have yielded a massive amount of brain connectivity data (Jones 2007). These data aid the characterization of neuroanatomical building blocks that constrain the functional limitations of brain networks (Sporns et al. 2000). However, the extent of the available brain connectivity data is so large, that it is challenging to analyze it without general statistical tools. To facilitate the analysis of neural networks, neuroscientist started to collate these data into so-called connectomes. Structural connectomes exist for different species like the nematode, the pigeon, the monkey, the rat and the mouse. Except for the nematode connectome, all connectomes existing today are partial connectomes in the sense that they do not contain complete connectivity information of the entire brain (Felleman and Van Essen 1991; White et al. 1986; Scannell et al. 1995; Dyhrfjeld-Johnsen et al. 2005; Shanahan et al. 2013; Bota et al. 2012; Schmitt et al. 2012; Oh et al. 2014; Burns and Young 2000; Sugar et al. 2011; van Strien et al. 2009). Such connectomes can then be mathematically dissected using graph analysis, which will elucidate objectively the specific organizational patterns and topological properties within the structural network that constrains its functional properties (Sporns et al. 2000; Shanahan et al. 2013; Schmitt et al. 2012; Bullmore and Sporns 2009; van den Heuvel and Sporns 2013; Fornito et al. 2012; Buckner and Krienen 2013).

In this study, we used graph analysis to examine the networks of the hippocampal formation and parahippocampal region (HF–PHR). The HF–PHR is engaged in spatial navigation and memory (Squire et al. 2007; Buzsaki and Moser 2013). A dominant view on hippocampus-dependent information processing suggests that information from the neocortex is relayed towards the hippocampus by two separate information-processing streams—A ‘what’ and a ‘where’ stream, concerning the perirhinal cortex–lateral entorhinal area and postrhinal cortex–medial entorhinal area, respectively (Burwell and Amaral 1998; Eichenbaum et al. 2012; Burwell 2000). Previously, we constructed an interactive wiring diagram of the rat HF–PHR (Sugar et al. 2011; van Strien et al. 2009) (http://www.temporal-lobe.com). The collated structural HF–PHR network is unique because we aimed to describe both the origin and termination position of connections at a high level of spatial detail. Whereas other cortical connectomes described connections at a macroscopic level, i.e. at the level of brain regions (i.e. CA1 or entorhinal cortex), the HF–PHR network divides each brain region along its three-dimensional axes such that a more specific volume of tissue can be designated as the site of origin or termination of a brain connection. Our approach at a more mesoscopic level provides an important next level of detail, since it is now known that a brain region can consist of functionally different parts that are found to be related to position along its three-dimensional axes (Brun et al. 2008; Henriksen et al. 2010; Jarrard et al. 2012; Oh et al. 2014). For instance, the projection from the entorhinal cortex to the hippocampus is topographically organized along the dorsoventral axis (Dolorfo and Amaral 1998). This topology affects the functioning of spatially tuned entorhinal grid cells. Grid cells found in the ventromedial part of the medial entorhinal area have much larger grid fields compared to their dorsolateral counterparts (Brun et al. 2008), which implies that distinct parts along the long axis of the hippocampus receive distinct information.

Here, we used graph analysis to determine the structural network properties of the HF–PHR of the rat. We classified the type of network and the significant network motifs. In addition, we identified hubs, distinct brain areas with a central position in the network, and modules, sets of brain regions cooperating in functional information processing. Our analysis indicated the presence of a rich club organization, in which highly connected brain areas are connected with other highly connected brain areas.

## Materials and methods

### Structural database of the rat HF and PHR

Our HF–PHR database is used to create the adjacency matrices for this study. The data entry procedure used to populate this database is described in detail in van Strien et al. (2009) and Sugar et al. (2011). In short, the HF–PHR connectivity database contains information about the existence of brain connections that were reported in peer-reviewed papers that describe results from anterograde and retrograde tract-tracing experiments as well as in intracellular filling studies. The focus of the database is limited to the HF and PHR in healthy, genetically un-altered, untreated adult rats (all strains) of both sexes. The tract-tracing method visualizes the course of axonal fibers and it distinguishes the direction of fibers (Lanciego and Wouterlood 2011; Otzas 2003). Unfortunately, in most cases it is neither possible to differentiate the excitatory or inhibitory nature of the projections, nor to objectively characterize the strength of the connections. The sites of origin and termination of HF–PHR connections were stored in the database. For this graph analysis study, only ipsilateral connections with clearly defined small injections sites confined within in a single brain area were selected from the database. In total, 117 studies that matched these criteria are included for the database, comprising almost 3,400 connections of the HF–PHR network (Sugar et al. 2011; van Strien et al. 2009)—see Suppl Data for references. The number of papers and reports that describe the existence of a connection varies (Suppl. Figure 3), which may partly reflect popularity of a certain sub area as a topic of study, rather than have meaningful value as a measure to estimate the confidence one may have in that a connection actually exists.

### Subdivision of the HF and PHR brain areas

The HF and PHR are commonly subdivided into smaller brain areas. The database follows the nomenclatures as explained by Cappaert et al. (2014). The HF brain areas (Suppl. Figure 1a, b) included in the database are the dentate gyrus (DG), Cornu Ammonis area 3 and 1 (CA3 and CA1, respectively), and the subiculum (Sub). Since the database was created, CA2 has received increased scientific attention, yet very little connectivity data exist today and, therefore, it was left out from the initial database. The PHR brain areas (Suppl. Figure 1 a, b) included in the database are the presubiculum (PrS), parasubiculum (PaS), the entorhinal cortex, which has a lateral (LEA) and a medial (MEA) subdivision, the perirhinal cortex (consisting of Brodmann areas A35 and A36) and the postrhinal cortex (POR).

An important feature of the HF–PHR database is that connections are described in terms that go beyond the subdivision of brain areas which allows greater accuracy in describing the origin and termination point of brain connections. To this end, for each brain region the position of origin and termination of a connection was described along each of its three anatomical axes (Suppl. Figure 1). Orthogonal to the surface of the cortex, we described a laminar dimension, representing each of the layers of a brain area according to the nomenclatures used in Cappaert et al. (2014). In the rostrocaudal dimension, either the rostrocaudal axis (for PER and POR) or proximodistal axis (for the HF brain areas, PrS and PaS) was defined and subdivided into three segments. Finally, in the dorsoventral dimension a septotemporal (for the HF brain areas, PrS and PaS), dorsoventral (PER and POR) or dorsolateral–ventromedial (dl–vm; for MEA and LEA) axis was defined and subdivided into three segments. A connection in the database is considered to have the highest level of spatial resolution if the position of the origin or termination of a projection is known along the subdivision of all three dimensions. For example, the origin or termination of a connection could be stored in the database as “the pyramidal cell layer (laminar dimension) of the proximal (rostrocaudal dimension), septal part (dorsoventral dimension) of area CA1”. Unfortunately, only a minority of the connections are reported at this high level of detail.

### Dataset preparation

After selecting connections from the database, the knowledge from matching, independent retro- and anterograde experiments was combined to determine the origin and termination of a connection more accurately. The resulting dataset with maximum spatial detail, i.e. with a breakdown of the brain areas into subregions along all three dimensions, resulted in a very sparsely populated connectivity matrix (see “Results”; Suppl. Figure 2). This issue was compromised by creating three separate sub-networks in which the connection matrix was shown for a single dimension, whereby the other two dimensions were collapsed (Suppl. Figure 2c). The first sub-network, called ‘rostrocaudal network’, consisted of connections within the rostrocaudal dimension. In the second sub-network, called ‘dorsoventral network’, we included all projections in which information in the dorsoventral dimension was available for the origin and termination of the connection. The dorsoventral axis in A35, A36 and POR was discarded, because there were too few reports on the origin or termination in this dimension of these anatomical regions, probably because this dimension is quite small. The third sub-network, called ‘laminar network’, comprised all projections in which layer information was available for the origin as well as the termination location, regardless of the presence or absence of information in the dorsoventral and/or rostrocaudal dimension.

### Graph analysis

#### Definition of nodes and edges

**Fig. 1:**
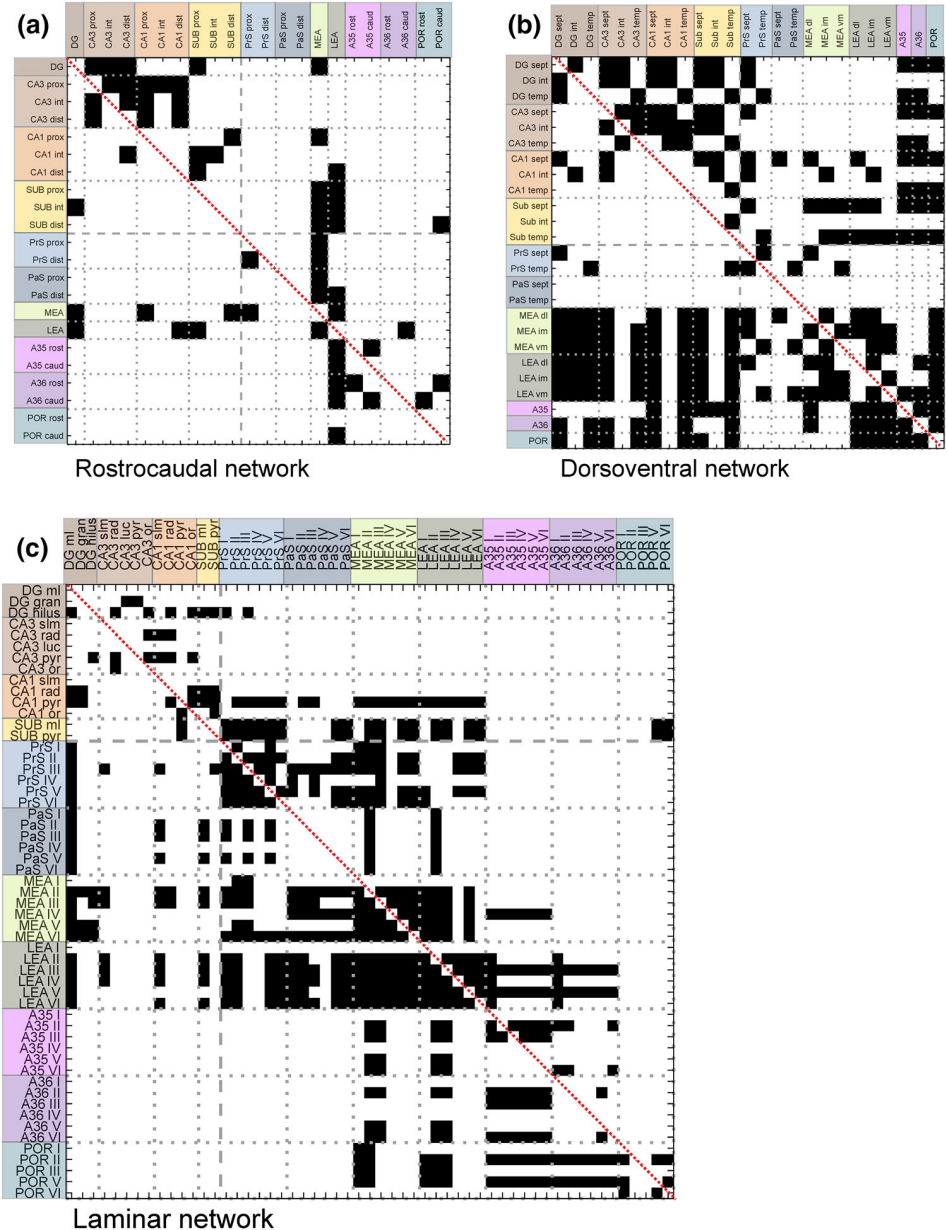
The directed, binary adjacency matrix of the rostrocaudal (**a**), dorsoventral (**b**) and laminar (**c**) network. The connections (or edges), presenting the axons or axon bundles, are indicated by *black squares*. The *white areas* represent projections that do not exist or are not documented yet. The self connections on the main diagonal (indicated with a *red line*) are excluded. The nodes represent the subareas of brain regions in the rostrocaudal dimension (**a**) or the dorsoventral dimension (**b**) and the layers of the brain regions (**c**). The nodes depicted on the *left* side represent the origin of a projection and the nodes on *top* represent the termination of a projection. The brain areas are *color* coded as follows: dentate gyrus (DG; *dark brown*), CA3 (*medium brown*), CA1 (*orange*) and subiculum (Sub; *yellow*), presubiculum (PrS; *medium blue*), parasubiculum (PaS; *dark blue*), entorhinal cortex (MEA; *light green* and LEA; *dark green*), perirhinal cortex [Brodmann areas (A) 35 (*pink*) and 36 (*purple*)] and the postrhinal cortex (POR; *blue green*). The *Roman numerals* indicate cortical layers. *caud* caudal, *dist* distal, *dl* dorsolateral part of the entorhinal cortex, *gran* granule cell layer, *im* intermediate dorsolateral–ventromedial part of the entorhinal cortex, *luc* stratum lucidum, *ml* molecular layer, *or* stratum oriens, *prox* proximal, *pyr* pyramidal cell layer, *rad* stratum radiatum, *rost* rostral, *sept* septal, *slm* stratum lacunosum-moleculare, *temp* temporal, *vm* ventromedial part of the entorhinal cortex

The nodes of the connectivity matrix consisted of subareas within an anatomically defined region which represented the origin or termination of projections. More specifically, nodes in the rostrocaudal network indicated the subregions of a brain area in the rostrocaudal dimension, for example, the rostral CA1 (Fig. 1a). In the dorsoventral network (Fig. 1b), the nodes represented subregions of a brain area in the dorsoventral dimension, for instance the septal part of DG (DG sept) or the dorsolateral part of the MEA (MEA dl). Nodes in the laminar network stood for a specific cortical layer (Fig. 1c), e.g. PrS layer I (PrS I) or the molecular layer of the CA1 (CA1 ml). Neuronal dendrites of cell bodies extend across layers, such that axonal inputs into different layers can synapse onto the same neurons.

The axons or axon bundles connecting pairs of nodes were the edges of the matrix. For example, the intermediate septotemporal part of the dentate gyrus (DG int) projected to the septal CA3 (CA3 sept; Fig. 1b). The connectivity matrices were binary and if a connection between a pair of nodes was reported in at least one tract-tracing paper, this was assigned 1. If no connection is reported or it was explicitly mentioned that there was no connection between two nodes, the value was set at 0. Projection strength and the excitatory or inhibitory nature of the connections were not incorporated in the network. The networks were directed graphs, resulting in asymmetric connectivity matrices. POR layer IV could not be included in the laminar network for a lack of incoming and outgoing projections. Self connections, represented on the diagonal of the connectivity diagram, were removed (Rubinov and Sporns 2010). Finally, this resulted in three matrices used for the graph analyses (Fig. 1a–c; Suppl. Data 1).

#### Graph theoretical measures

Graph measures for binary, directed graphs were computed using the Brain Connectivity Toolbox ((Rubinov and Sporns 2010) (http://www.brain-connectivity-toolbox.net/). A brief description of the computed metrics includes:

*Degree and density* The degree quantifies the number of all connections (edges) from one node to the remainder of the network. The degree of a node reflects the importance of this node in the network. Brain regions with a high degree are interacting with a high number of other brain regions. The node degree was separated into indegree, including the incoming (afferent) connections and outdegree, including the outgoing (efferent) connections of a region. Topological neighbors were defined as nodes that have a direct connection with a particular node. The density is the proportion of edges that exist relative to the number of potential edges of a network. The density gives an impression of how well connected a network is (Kaiser 2011).

*Distance, characteristic path length and diameter* The distance between two nodes is the minimal number of edges that have to be crossed to connect two nodes. Note that the measure ‘distance’ does not reflect the physical distance between nodes. The characteristic path length of a network is the mean distance for all node pairs. The path length is a measure of connectivity in the network—a high characteristic path length implies low global network connectivity, whereas a low path length indicates high connectivity. The diameter of the network is the maximum of the shortest path between two nodes (“the longest shortest path”).

*Clustering coefficient* The clustering coefficient quantifies the mean fraction of the actual connections between the neighbor’s nodes and the maximum possible number of connections between these neighbors (Watts and Strogatz 1998). The cluster coefficient depicts the ability of the neighbors of a node to connect with each other. The mean clustering coefficient over all nodes characterizes the prevalence of clustering in the network.

### Small-world organization

Watts and Strogatz (1998) described small-world network models as networks with high clustering and a small characteristic path length compared with the same parameters defined in 100 random null models (see below). Such a network organization provides a balance between functional integration and segregation (Sporns and Honey 2006). Values of path length and clustering coefficient were considered significantly different when they differed more than one standard deviation (SD) from the mean of the respective values of the 100 random null models. Regular networks have high path length and high clustering coefficient and random networks are characterized by short path length and almost no clustering (Watts and Strogatz 1998).

*Motifs* Structural motif analysis divides the network into characteristic small, unique anatomical building blocks of a certain number of nodes (Milo et al. 2002; Sporns and Kotter 2004). Motifs of *M* = 3 nodes can be classified into 13 distinct structural motif classes and the motif count represents the number of times a certain motif class occurs in the investigated network. As a reference, the motif frequency spectra were computed for random null models. A motif count was considered significantly higher when the *z* score for a certain motif was >5.0, *p* < 0.0001 (Sporns and Kotter 2004). Next, the motif participation number, the number of instances a node participates in a specific motif class, is determined and compared with the motif participation number found in the random null model (Sporns and Kotter 2004).

*Modules* Modules are groups of nodes with a high connection density within the module and low connection density with other modules. A spectral modularity optimization algorithm that defines sets of nodes which have a maximized number of within-group edges and a minimized number of between-group edges was used for the decomposition of the network into non-overlapping, non-hierarchical modules (Leicht and Newman 2008). The modularity *Q* score, which represents the fraction of connections in a module relative to the expected fraction of connections in a module based on chance, is optimized to obtain the modules of the current network. The modules were constructed at least 50 times and the module set with the highest modularity *Q* score was selected.

*Participation index* The participation index (*P*_i_) quantifies how the connections of a node are distributed across all modules. A *P*_i_ close to 1 is perceived when all connections of a node are uniformly distributed to all modules; *P*_i_ is close to 0 if all the connections stay within the module to which this node belongs.

*Network hubs* Hubs are brain regions that occupy a central position in the network and hubs are defined as nodes that interact highly with other nodes. In this study, hubs were classified into three groups, based on degree and the value of *P*_i_ (van den Heuvel and Sporns 2011, 2013). The degree is classified into two categories—high and low degree. High degree nodes have a degree which is at least one standard deviation above the mean total degree, while low degree nodes are the remaining nodes, i.e. those that were not classified as high degree nodes. The first group contained the non-hub connectors, based on their low degree. The second group included the provincial hubs. These provincial hubs are connecting nodes within their own modules and are defined as nodes with a high degree and *P*_i_ ≤ 0.3. The third group encompassed the connector hubs, which link different modules. Connector hubs were defined as a high degree and a *P*_i_ > 0.3.

*Rich club* A group of high degree nodes which are densely connected among themselves, is called a rich club phenomenon (Colizza et al. 2006). A rich club was detected by systematically removing nodes from the network according to their total node degree, starting from the lowest degree to the (second) highest degree. First, the degree of each node was calculated and subsequently all nodes with a degree ≤*k* are removed, resulting in a subgraph with a degree >*k*. The rich club coefficient for this subgraph, *φ*(*k*), was the connection density of the subgraph. The *φ*(*k*) was defined for all *k*’s and this resulted in the rich club curve (Suppl. Figure 5). As a reference, *φ*(*k*) was also calculated for 1,000 random null models (see below), resulting in *φ*_random_(*k*)—the mean of this model. The normalized rich club coefficient (*φ*_norm_) was derived by the ratio of *φ*(*k*) over *φ*_random_(*k*). A rich club is potentially present if *φ*_norm_ is significantly >1 over a range of *k*’s (van den Heuvel et al. 2012; van den Heuvel and Sporns 2011), and we checked if the *φ*(*k*) is outside the 95 % interval of the *φ*_random_(*k*). All tests used a Bonferroni-correction.

*Intermediate node* Two nodes that are directly connected (distance = 1) can exchange information directly (Fig. 5a, black squares). However, nodes that are not directly connected may influence each other via an intermediate node (Fig. 5b, inset). For instance, PrS layer II was not directly projecting to the DG granular cell layer (Figs. 1c, 5a), but via intermediate node MEA layer II, PrS layer II can influence the DG granular cell layer. To examine which nodes facilitate the connection between not-directly connected pair of nodes, we modified the ‘flow coefficient’ measure of Honey et al. (2007) into a measure called ‘intermediate node’. The intermediate node quantifies the number of times a specific node is the middleman between a pair of nodes with distance 2, in the configuration of Motif 2 (*M* = 3, Fig. 5b, inset).

**Fig. 2:**
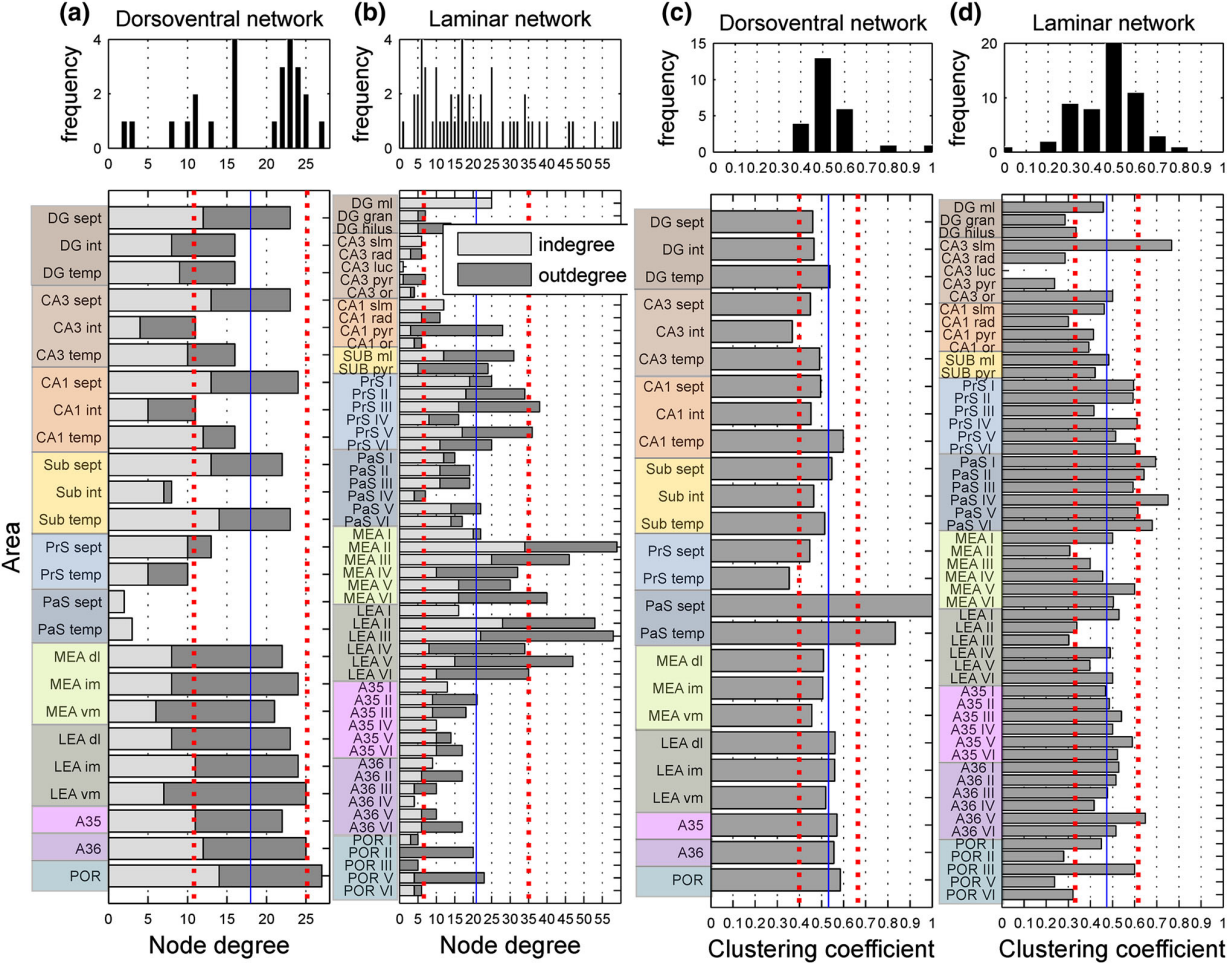
The in- and outdegree specified per node for the dorsoventral (**a** bottom) and laminar network (**b** bottom). The *blue lines* mark the mean degree, the *red dotted lines* mark the mean plus or minus the standard deviation (SD), revealing the nodes with a significantly high or low node degree. The figures in the *top* represent the distribution of the node degree. The cluster coefficient specified per node for the laminar network (**c** *bottom*) and the dorsoventral network (**d** *bottom*). The *blue lines* mark the mean cluster coefficient; the *red dotted lines* mark the mean plus or minus the SD, revealing the nodes with a significantly high or low cluster coefficient. The figures in the *top* represent the distribution of the cluster coefficient. For abbreviations, see legend of Fig. 1

First, node pairs with distance 2 were identified in the distance matrix (Fig. 2a) and subsequently all possible nodes were identified that can connect the node pairs with distance 2 to form a unidirectional sequence of nodes the top node in Motif 2 (Fig. 5). These interlinking nodes were named the intermediate nodes. For visualization purposes, the nodes at the start and the end of the chain were lumped into four predefined anatomical sets: set (1) all four areas of the HF, set (2) PrS and Pas set (3) the LEA and MEA, and set (4) PE R and POR.

*Random null model* A random null model was used for statistical reference. The edges of the original connection matrix were randomly rewired 100 times using a Markov random switching algorithm (Maslov and Sneppen 2002). This randomization algorithm preserves the network’s size, density and in- and outdegree distribution. Each random null model was generated 100 or 1,000 times and then our HF–PHR network was statistically compared with the random null model.

## Results

### Global description of HF–PHR network

A network at macroscale was created with 11 nodes representing the main brain regions of the HF–PHR (Suppl. Figure 3D), revealing a very densely connected network (density = 96 %). To obtain a network with a lower density, a network at a more mesoscopic level was created. The volume of each of the 11 HF–PHR brain areas was subdivided along their three-dimensional axes and this resulted in a network with a total number of 327 nodes (Suppl. Figure 2). The nodes of the brain areas PrS, PaS, A35, A36 and POR were completely devoid of in- and outgoing connections. This sparsity is caused by an absence of detailed reports in the literature, i.e. the exact origin and termination location in all three dimensions is not described. Even after removing the 70 isolated nodes, the matrix showed a connection density of just 0.75 %. To reduce the number of leave nodes and at the same time preserving all HF and PHR brain areas for the analysis, we decomposed the total network into three sub-networks—(1) the rostrocaudal network, (2) the dorsoventral network and (3) the laminar network (Suppl. Figure 1).

### Rostrocaudal network

The rostrocaudal network (Fig. 1a) consisted of 22 nodes, 58 edges and a connection density of 12 %. The mean degree was 5.2 in a range from 1 to 16. The network had a diameter of 5 and a path length of 2.4. The number of times each edge reported in the literature for the rostrocaudal network was high, a mean of 15.9 reports per edge was observed (Suppl. Figure 3a).

### Dorsolateral network

The HF–PHR dorsoventral network (Fig. 1b) consisted of 25 nodes and 225 edges, ensuing in a connection density of 36 %. The mean degree, sum of the in- and outdegree, of the dorsoventral network (Fig. 2a) was 18.0 in a range from 2 to 27. The POR was discerned as a brain area with a high total degree of 27. The dorsoventral network had a path length of 1.62 and a diameter of 4. The mean number of reports per edge was 6.1 (Suppl. Figure 3b).

**Fig. 3:**
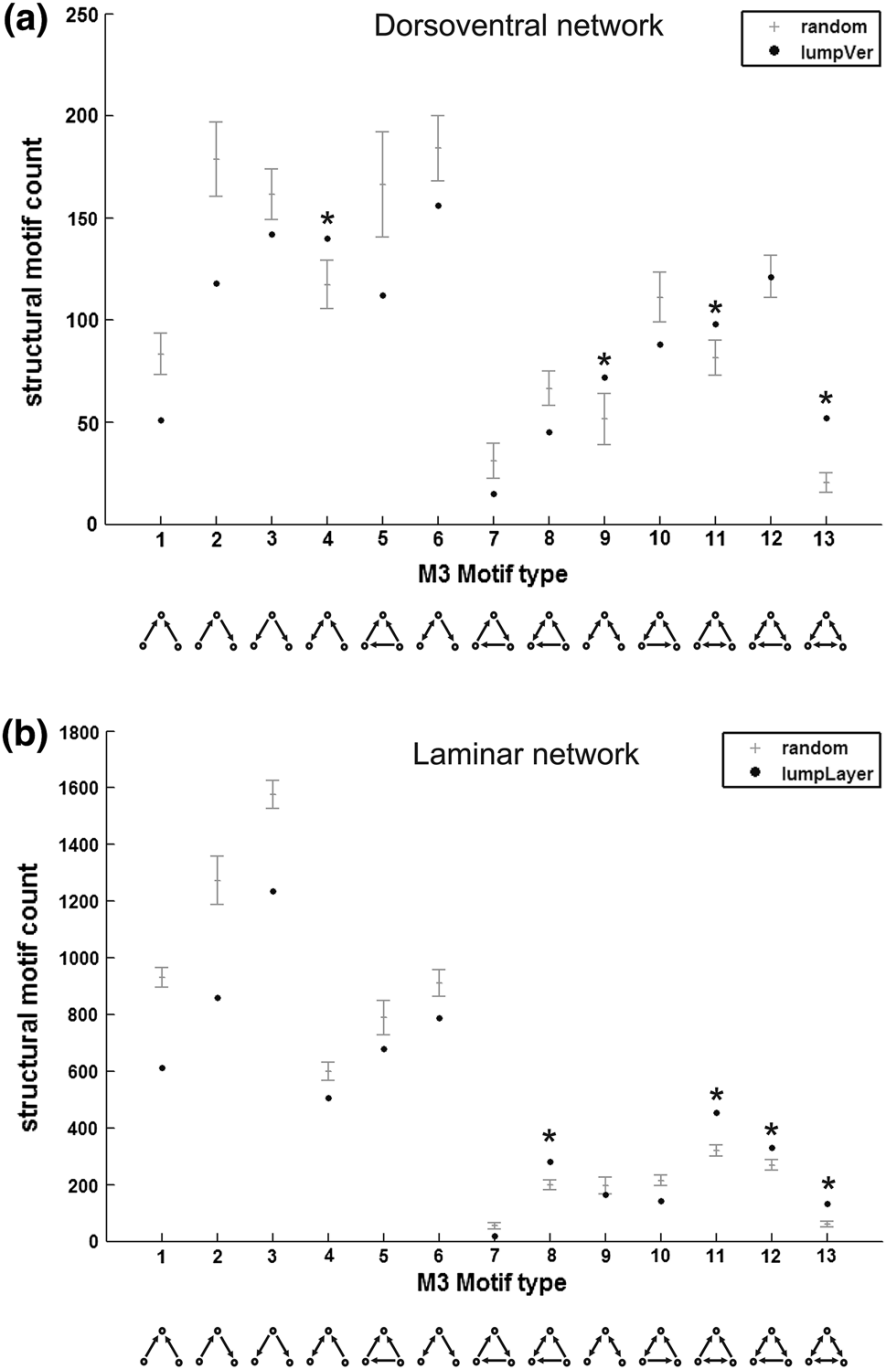
Structural motif analysis of the dorsoventral (**a**) and laminar network (**b**). The frequency of the *M* = 3 directed motifs is indicated with a *black circle* and the motif counts of the random null models are represented by the mean ± standard deviation (*grey lines*). *Asterisk* indicate motifs with significantly increased occurrence over random networks

To categorize the global network properties, we calculated the cluster coefficient (Fig. 2c) and the characteristic path length of the HF–PHR network. The mean cluster coefficient of the dorsoventral network was 0.53, which was higher compared with the random null model of the dorsoventral network (0.49 ± 0.02—mean ± SD). The path length of the dorsoventral network (1.62) was larger than that of the random network (1.58 ± 0.01). The *M* = 3 motif types 4, 9, 11 and 13 occurred significantly more often in the dorsoventral network than in the random null model (Fig. 3a). The motif participation number revealed that certain nodes participated more strongly in certain motif classes. The intermediate dorsolateral–ventromedial MEA, the ventromedial MEA and the intermediate septotemporal CA1 contributed strongly to Motif 4. The temporal Sub was highly involved in Motif 9 and 13, the intermediate dorsolateral–ventromedial MEA and LEA were considerably involved in Motif 11 and the POR participated substantially in Motif 13 (data not shown).

**Fig. 4:**
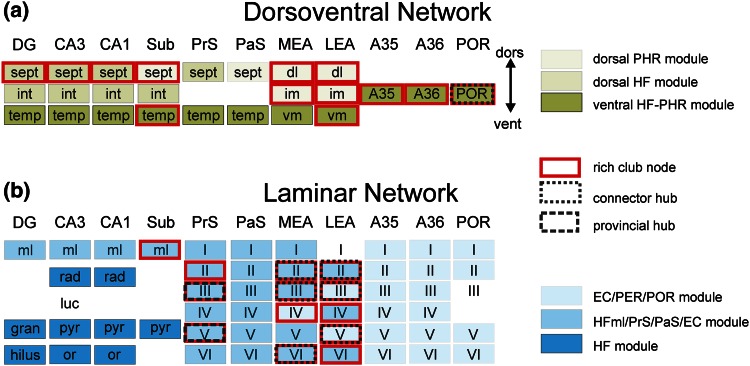
Overview of the nodes of the dorsoventral (**a**) and laminar network (**b**). The modules are represented in *color*, the members of the rich club are represented in *red* and the connector and provincial hubs are outlined with a *black box*. For abbreviations, see legend of Fig. 1

Three distinct modules were detected in the dorsoventral network with a modularity *Q* score of 0.15 (Fig. 4a; Suppl. Figure 4a). The largest module, ‘ventral HF–PHR’, comprised a total of twelve temporal areas, including the temporal areas of the hippocampal formation, PrS and PaS, the ventromedial LEA and MEA, the intermediate LEA and PER and POR. The second largest module, ‘dorsal HF’, consisted of eight nodes, incorporating the septal and intermediate septotemporal areas of DG, CA3 and CA1, the intermediate septotemporal Sub and septal PrS. The smallest module, ‘dorsal PHR’, included the remaining five nodes—the dorsolateral MEA and LEA, the intermediate dorsolateral–ventromedial MEA, the septal PrS and Sub.

The POR was the only node in the dorsoventral network that had a high node degree and based on its intermediate participation coefficient of 0.54, POR was distinguished as a connector hub (Fig. 4a).

A rich club community of thirteen nodes was discerned in the dorsoventral network (Suppl. Figure 5a; Fig. 4a). A significant increasing normalized rich club coefficient was detected for node degree *k* < 16 < 21 and the *φ*(*k*) was significantly outside the 95 % interval of the *φ*_random_(*k*). The septal parts of the HF areas and the dorsolateral parts of the MEA and LEA were detected as rich club nodes. Moreover, the temporal Sub and the ventromedial part of the LEA, the intermediate dorsolateral–ventromedial band of the EC, and the A35, A36 and POR were rich club members.

### Laminar network

The laminar network consisted of 55 nodes and 573 edges (Fig. 1c; Suppl. Figure 3e—the Connectome Viewer; Gerhard et al. 2011), resulting in a connection density of 19 %. On average, 3.4 occurrences were reported per edge (Suppl. Figure 3c). Relatively high reporting rates were found for the entorhinal–hippocampal connections (around 750 reports for the ingoing and outgoing connections). The PrS connections were most frequently reported on in the rat connectivity literature describing the laminar topology. This is based on the outstanding single cell filling study of Honda and colleagues (Honda et al. 2011). The mean degree, the sum of the in- and the outdegree (Fig. 2b) of the laminar network was 20.8 and the degree ranged substantially from 1 to 59. PrS layers III and V and entorhinal layers MEA II, III, VI and LEA layers II, III and V were high degree nodes. The laminar network had a path length of 2.11 and a diameter of 5.

The mean cluster coefficient (Fig. 2d) of the laminar network (0.47) was higher compared with the mean cluster coefficient of the random null model (0.42 ± 0.01). Also, the path length of the laminar network (2.11) was higher than the path length of the random null model (1.89 ± 0.02).

The local connectivity analysis for directed *M* = 3 motifs of the laminar network revealed four significant motifs (motif type 8, 11, 12 and 13) with *z* score >5.0 in comparison with the random null model (Fig. 3b). The hippocampal pyramidal cell layer of CA1 and Sub participated particularly in Motif 8 while the PHR regions contributed more strongly in motifs 11, 12 and 13.

Five modules, with a modularity *Q* score of 0.23, were detected in the laminar network (Suppl. Figure 4b; Fig. 4b). The largest module, consisting of 24 nodes, was labeled ‘HFml/PrS/PaS/EC’. This module contained the molecular layers of the hippocampal fields, all PrS and PaS layers, all layers of the MEA—except layer IV and LEA layers II, IV and VI. The second module, ‘EC/PER/POR’, consisted of 19 layers: all layers of PER and POR and three EC layers—MEA IV, LEA III and V. The third module, ‘HF’, confined the layers of the hippocampal formation, except for the molecular layers of HF. The small fourth and fifth module consisted of LEA layer I and POR layer III, and CA3 stratum lucidum, respectively.

The participation index of the eight high degree nodes in the laminar network was calculated to define if these nodes could be characterized as provincial or connector hubs (Fig. 4b). MEA layers II, III and VI and LEA layers II, III and V were classified as connector hubs in the laminar network, based on their high degree and *P*_i_ > 0.3. PrS layers III and V were categorized as provincial hubs, with a high degree node and *P*_i_ ≤ 0.3.

The laminar network displayed a rich club organization (Suppl. Figure 5b; Fig. 4b). For the node degrees 7–30, there was a normalized rich club coefficient >1 and the rich club coefficient of the random network was significantly below the rich club coefficient of the laminar network. A total of thirteen rich club nodes were distinguished and the majority of these nodes were situated in the parahippocampal region. The rich club is dominated by nine nodes located in the layers of the LEA and MEA. The only HF rich club node was the molecular layer of the Sub.

**Fig. 5:**
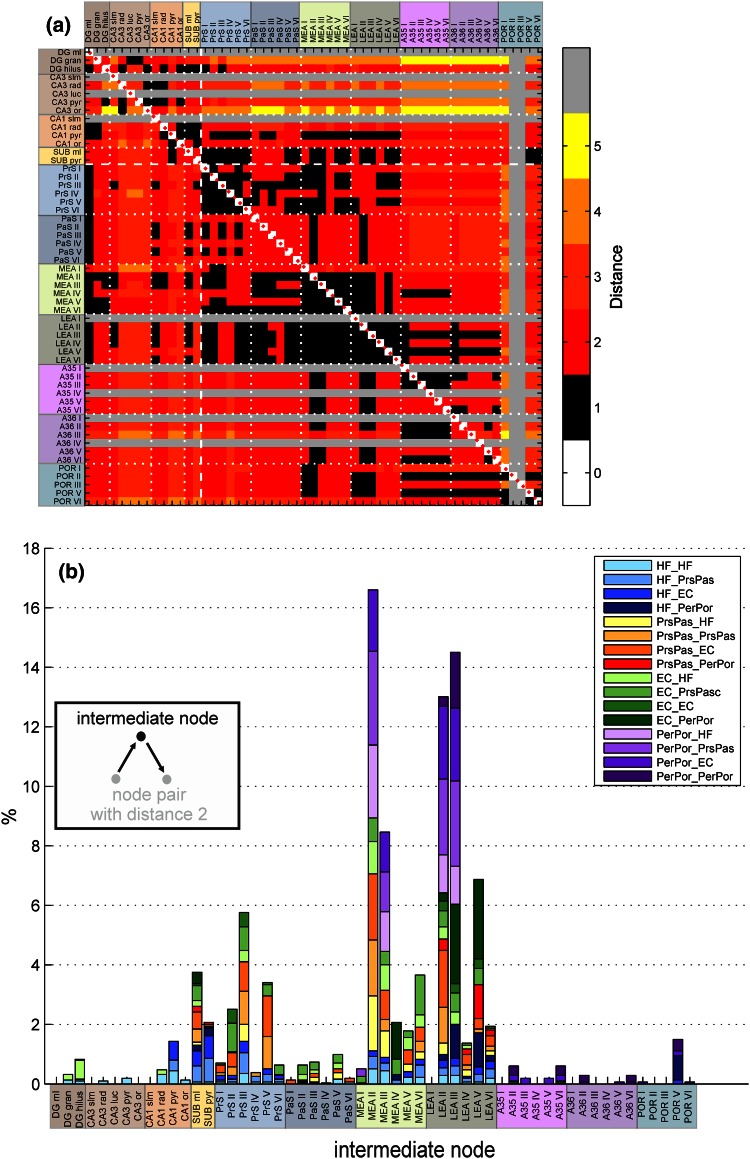
**a** Distance matrix of the laminar network. The *color scale* indicates the smallest number of steps between pairs of nodes. **b** The percentage times each node of the laminar network are located in the intermediate position of two not-directly connected nodes (see *inset*). The origin and termination of the not-directly connected node pairs are clustered into four groups—the node of the subregions of the hippocampal formation (HF), the PrS and PaS (PrSPaS), the medial and lateral entorhinal cortex (EC) and the perirhinal and postrhinal cortex (PERPOR). For abbreviations, see legend of Fig. 1

The intermediate node was determined for the laminar network (Fig. 5). No direct projections from PrS and PaS to PER and POR were found (Fig. 5a), but information transfer between these areas could still occur via the LEA layers (Fig. 5b, red bar). If information flow from PER/POR to the other HF–PHR areas would use two steps, the MEA and LEA would serve as intermediate nodes. Moreover, LEA might also play an important role in the information flow from PER/POR via LEA back to PER/POR. PER/POR were not directly projecting to the HF and PrS/PaS region (Fig. 5a). For information to flow to these areas, the route via the superficial layers of MEA and LEA is most likely (Fig. 5b). Based on the frequency the superficial layers of the MEA and LEA were positioned as intermediate nodes, suggested that these nodes play a dominant role in the information transfer between several HF–PHR areas.

## Discussion

The rat HF–PHR structural connections were investigated with graph analysis to reveal principles of the network organization. In contrast to other brain networks, which all showed a small-world organization with high clustering and low path length (Sporns and Zwi 2004; Oh et al. 2014; Schmitt et al. 2012; Shanahan et al. 2013; Watts and Strogatz 1998), the HF–PHR network revealed a high cluster coefficient and a relatively high path length, suggesting a more regularly organized network. Nevertheless, some small-world features, like modularity, rich club and hub nodes, were present in the HF–PHR network. Regular networks with high clustering and high path length have a highly ordered structure. Anatomically, this means that local connections prevail and that distant brain areas can only be reached via a high number of intermediate areas. In contrast, in a small-world network, with high clustering and low path length, distant brain areas can be reached within a small number of steps. A possible explanation for such a network architecture with a high clustering and path length might be found in the fact that the HF–PHR belongs to a phylogenetic older part of the brain—the limbic system—which could be differently organized compared with newer parts, as the neocortex. However, the reticular formation in the brain stem, the phylogenetically oldest part of the mammalian brain, also showed a small-world network structure (Humphries et al. 2006; Coolidge and Wynn 2009). A more plausible explanation points in the directions of the anatomical scale considered in the HF–PHR network. Compared with other mammalian connectomes of the cat and the macaque cortex (Felleman and Van Essen 1991; Scannell et al. 1995) the nodes in our HF–PHR network represent smaller anatomical elements. Therefore, the number of nodes in our network is much higher than the five or six nodes used to represent the same anatomical areas in the cat or macaque connectome. Furthermore, the different subregions of the HF–PHR are probably all involved with functionally similar memory processes. In all, our finding of a high clustering and relatively high path length appears to be consistent with the idea of Bullmore and Sporns (2009, 2012) that specialized processes are performed within a small number of anatomically adjacent brain regions with a high clustering, while distributed processes tend to use a more widespread network of brain areas.

Structural motifs may tell something about the particular information-processing function that a part of the network fulfils. In the rat HF–PHR network, we found four motif types with increased occurrence over random networks in the laminar and dorsoventral network. This is unique compared with other brain networks, which showed predominantly an increase in one specific motif class in mammalian network (Shanahan et al. 2013; Sporns and Kotter 2004; Schmitt et al. 2012) and two motifs in the nervous system of the invertebrate nematode *C. elegans* (Sporns and Kotter 2004). A commonly reported motif class is type 9, which is a chain of reciprocal connections, but no connection between start and end of the chain. When we compared this commonly occurring motif with our increased motif types, we saw that all discovered HF–PHR motifs had at least one reciprocal connection, which may serve feedback loops, and most motifs were converging onto one node, suggesting feed-forward loops.

### Challenges and possible refinements

The network was mainly based on published tract-tracing data and this leads to certain limitations in the analysis that occur specifically due to limitations of this experimental technique. Graph analysis of this network included two notable challenges. The first challenge was how to estimate the reliability of the existing and absent connections. For instance, the absence of projections between two areas can occur because—(1) the projection does not exist in the brain, (2) the projection does exist, but is not yet documented or (3) the combination of the connectional information into sub-networks led to a loss of data. This leads to some surprising voids in the adjacency matrices. For instance, no intrinsic connections within the Sub and PaS were represented in the laminar network, although, based on the literature, they exist (Cappaert et al. 2014; see also Suppl. Figure 3d). Moreover, there are connections reported between POR and the hippocampal areas DG, CA3, CA1 and Sub in the literature (Suppl. Figure 3d). These connections were discernible in the dorsoventral network (Fig. 1b), while the projections were absent in the laminar network (Fig. 1c). Based on the tract-tracing literature, connections exist between almost all brain areas (Suppl. Figure 3d). Unfortunately, many reports of brain connectivity describe the location regarding the origin and termination of the connections in too limited amount of detail. This indicates that scientists underestimate the (importance of the) extensiveness of the network, which limits the interpretation of the network using graph analysis. A measure of reliability that indicates if projections actually exist could partly be based on the frequency with which a connection was reported in the literature. However, since this frequency also depends on the scientific attention that a certain brain area or its connections have received, this reliability value can only be used in combination with other evidence and has limited value on its own.

Second, it was not feasible to analyze the undivided database in all three dimensions within one complete dataset. The nodes of four out of the eleven HF–PHR brain regions were not connected to the network, because the origin and termination of most connections are only described in one or two dimensions in the literature. This gave rise to loss of data and the total three-dimensional dataset turned out to be too sparse. This was compromised by creating three separate sub-networks, in which the connections were presented for one dimension and while the other two dimensions were collapsed. Combining information into three complementary sub-networks gave the opportunity to study the network in one plane independently. Although in this case, the exact interplay of the connections within the three-dimensional space simultaneously remained unexamined.

A possible refinement will be the classification of the connections into excitatory, inhibitory, or modulatory. The tract-tracing technique disregards the type of neurotransmitter and its effect on the postsynaptic element. Combining tract-tracing data with immunohistochemistry and/or electrophysiology would make it possible to functionally interpret the connections (van Haeften et al. 1997; Jinno 2009; Somogyi and Klausberger 2005). Unfortunately, most anatomical papers do not provide such information. Therefore, the current HF–PHR network did not differentiate between excitatory or inhibitory projections. The network just scored the presence of an anatomical connection, indicating possible pathways for information exchange. Another refinement concerns the connection strength of the projections. Some anatomical literature does report subjective descriptions of connectional density, but such subjective reports are difficult to compare and quantify without extensive re-analysis of the original data (Kennedy et al. 2013). Besides, the strength of neuronal projections can be altered according to experience and learning as well (Fu and Zuo 2011). We decided to represent the connections of the HF–PHR network in binary adjacency matrices, presuming that all connections have the same influence. Finally, increasing the number of areas involved in the network could change the measures, as additional connections may change degree, path length and other measures.

All in all, these challenges and potential refinements indicate that translating anatomical connections into functional properties is complicated; the anatomical network only provides a framework for possible functional dynamics to occur and one has to be careful with the interpretation of the ‘no connection’ data, because ‘no data’ do not necessarily mean ‘no connection’.

### HF–PHR modules and the two-stream memory model

The modularity analysis partitioned the nodes of the laminar and dorsoventral network into three modules with a substantial number of nodes. The modular organization of the HF–PHR network differed slightly from the clusters Burns and Young (2000) discovered in their limbic network, using non-parametric clustering analysis. They found that, amongst other limbic areas, the HF areas and the entorhinal cortex were closely associated within one cluster; the PrS, PaS and POR were clustered into a second group. The PER was positioned in a third cluster, which also included anatomical areas outside the HF or PHR. This slightly different arrangement of the modules could be produced by the difference of the initial data in the connectome of Burns and Young which included connections to all areas of the limbic system, but with much less detail.

The HF and the PHR are components of the medial temporal lobe and play an important role in declarative memory and all the brain areas have their own role in the encoding, retrieval and consolidation of memory (Squire et al. 2004; Eichenbaum et al. 2012). Commonly, two information-processing streams through the PHR to the HF are distinguished—the POR and MEA are clustered in the spatial or ‘where’ stream, while the PER and LEA are collaborating in the non-spatial or ‘what’ stream. These two streams are supposed to have little or no *functional* connections between them. Finally, the information from the two PHR streams enters the HF, where it is further processed (Burwell and Amaral 1998; Eichenbaum and Lipton 2008; Eichenbaum et al. 2012; Deshmukh and Knierim 2011; Hargreaves et al. 2005; Bannerman et al. 2002; Knierim et al. 2006; Eichenbaum and Fortin 2005; Ranganath and Ritchey 2012). In the discussion below, we will consider if the modularity analysis of the HF–PHR network supported the current state of the art view of memory processing, as nodes assigned to one module are thought to contribute to a common function (Sporns and Zwi 2004).

The HF module of the laminar network contained all HF areas and their layers, except the molecular layer, suggesting that most HF connections were intrinsic and the molecular layers of the HF were grouped in the same module as the EC, PrS and PaS. Both findings support the view that the HF is functioning as a separated structure in the HF–PHR network, receiving converging spatial and non-spatial related information from the MEA/PrS/PaS and LEA, respectively (Eichenbaum et al. 2012). The grouping of the MEA, PrS and PaS into one module fits the two-stream model as well, since these brain areas are all three associated with spatial functioning (Boccara et al. 2010; Solstad et al. 2008; Taube et al. 1990). The presence of POR (‘where stream’) together with LEA and PER (‘what’ stream) in the same module and the clustering of LEA and MEA in one module, suggest at least possible anatomical connections between the ‘where’ and ‘what’ streams. Although several physiological findings in the literature support the two-stream model (Sargolini et al. 2006; Solstad et al. 2008) other reports are inconsistent. More and more reports claim that the LEA is also involved in spatial information processing (Van Cauter et al. 2013; Deshmukh and Knierim 2011; Tsao et al. 2013) and MEA contributes to item recognition memory as well (Hunsaker et al. 2013). This may request a refinement of the two-steam memory model, including interconnectivity between PER and POR and interaction between PER and MEA, and POR and LEA. However, care has to be taken; since our laminar network is probably incomplete due to lack of data and functional connections may be different from anatomical ones.

The modularity analysis of the dorsoventral network resulted in a module representing all the ventral parts of the HF and the PHR areas. The dorsal parts were split into two modules—a module with dorsal HF areas and one with the dorsolateral areas of the EC. The composition of these dorsoventral modules might imply different functions for the ventral and dorsal parts of the HF–PHR network. A dorsal–ventral separation was already proposed in 1998 by Moser and Moser as the dorsal HF is engaged in spatial functioning, whereas the ventral HF is involved in item recognition (Moser and Moser 1998). Furthermore, the place-field size of the place cells is increasing from the dorsal to the ventral pole of the HF (Jung et al. 1994; Maurer et al. 2005; Kjelstrup et al. 2008) and this dorsoventral gradient is reported for the grid size of the MEA grid cells as well (Brun et al. 2008). These findings lead to the idea of a double dissociation between two parallel information streams, a dorsal and a ventral one. The composition of the modules of the dorsoventral network supported in some way the idea of parallel systems, but mainly for the ventral brain areas. This discrepancy could be caused by the relatively low Q modularity value of the dorsoventral network, indicating high connectivity between modules which indicates an incomplete dissociation between the parallel streams.

### The role of the entorhinal cortex

Most of the nodes discerned with hub and rich club analyses in the laminar network were situated in the EC, predicting an important role of this brain region within the HF–PHR. The anatomical subdivisions of the EC were remarkably clustered together with both HF areas and PER/POR within the laminar and dorsoventral modules, taking a central position within the network and indicating a bridge function between the HF and the PER and POR. The intermediate node analysis implied that the connector hubs of the superficial layers of LEA facilitate the indirect connection between PER/POR and the PHR network, probably integrating information between modules. MEA layer II, also identified as a connector hub, showed the highest percentage of intermediate node positions, mainly between the HF, PrS/PaS and LEA/MEA subcategories, but not from and to the PER and POR.

It has been suggested that brain disorders affect high-cost hub nodes of the network (Bullmore and Sporns 2012; Crossley et al. 2013; van den Heuvel and Sporns 2013). This was corroborated by our study. The EC is a brain structure involved in several pathological conditions. The EC has been noted to show neuron and volume loss in patients with Parkinson’s disease, schizophrenia, early Alzheimer’s disease and aging (de Leon et al. 2001; de Toledo-Morrell et al. 2000; Braak and Braak 1985, 1991). Although the emphasis is generally put on the deterioration of the HF, the EC is affected in MTL epilepsy as well. Neuroimaging and neuroanatomical human studies have suggested that the volume of the EC is significantly reduced (Bartolomei et al. 2005; Bernasconi et al. 2001) and that damage can mainly be attributed to cell loss in superficial layers of the MEA (Du et al. 1993). These superficial EC layers are essential in the network, since they were assigned as connector hubs and important intermediate nodes. This suggests that loss of these cell layers seriously deteriorates the information exchange between other HF–PHR areas and proving the theory that high-cost hub nodes are first attacked during brain disorders.

Graph theoretical analysis of the HF–PHR network showed that this network had a high clustering and relatively high path length and several modules and hub nodes were present. It is suggested to reconsider the strict segregated of the ‘where’ and ‘what’ streams in the two-stream memory model, as anatomical interconnections between POR and PER, and MEA and LEA are present. Moreover, our findings implied that the entorhinal cortex occupies a central position in the HF–PHR network.

## Electronic supplementary material

Below is the link to the electronic supplementary material.

Supplementary material 1 (PDF 1686 kb)

Supplementary material 2 (DOCX 46 kb)
