# Supplementary material for: Graph analysis of the anatomical network organization of the hippocampal formation and parahippocampal region in the rat
Source: Brain Struct Funct. 2015 Jan 25;221(3):1607–21. doi: 10.1007/s00429-015-0992-0 (PMC4819791; doi:10.1007/s00429-015-0992-0)
Supplement: Supplementary file 1 — Supplementary material 1 (PDF 1686 kb) [file 429_2015_992_MOESM1_ESM.pdf]

Supplementary figures of:

Graph analysis of the anatomical network organization of the hippocampal formation and parahippocampal region in the rat

F.Z.M. Binicewicz[1], N.M. van Strien[2], W.J. Wadman[1], M.P. van den Heuvel[3,4], N.L.M. Cappaert[1]

[1] Swammerdam Institute for Life Science - Center for Neuroscience, University of Amsterdam, Amsterdam, The Netherlands

[2] Kavli Institute for Systems Neuroscience and Centre for Neural Computation, Norwegian University of Science and Technology, Trondheim, Norway

[3] Department of Psychiatry, University Medical Center Utrecht, Utrecht, The Netherlands

[4] Brain Center Rudolf Magnus, University Medical Center Utrecht, Utrecht, The Netherlands

Corresponding author:
N.L.M. Cappaert, PhD
Swammerdam Institute for Life Sciences – Center for Neuroscience
University of Amsterdam
Science Park 904, room C3.266
1098 XH Amsterdam
The Netherlands
email: N.Cappaert@uva.nl
tel.: + 31 20 525 7625

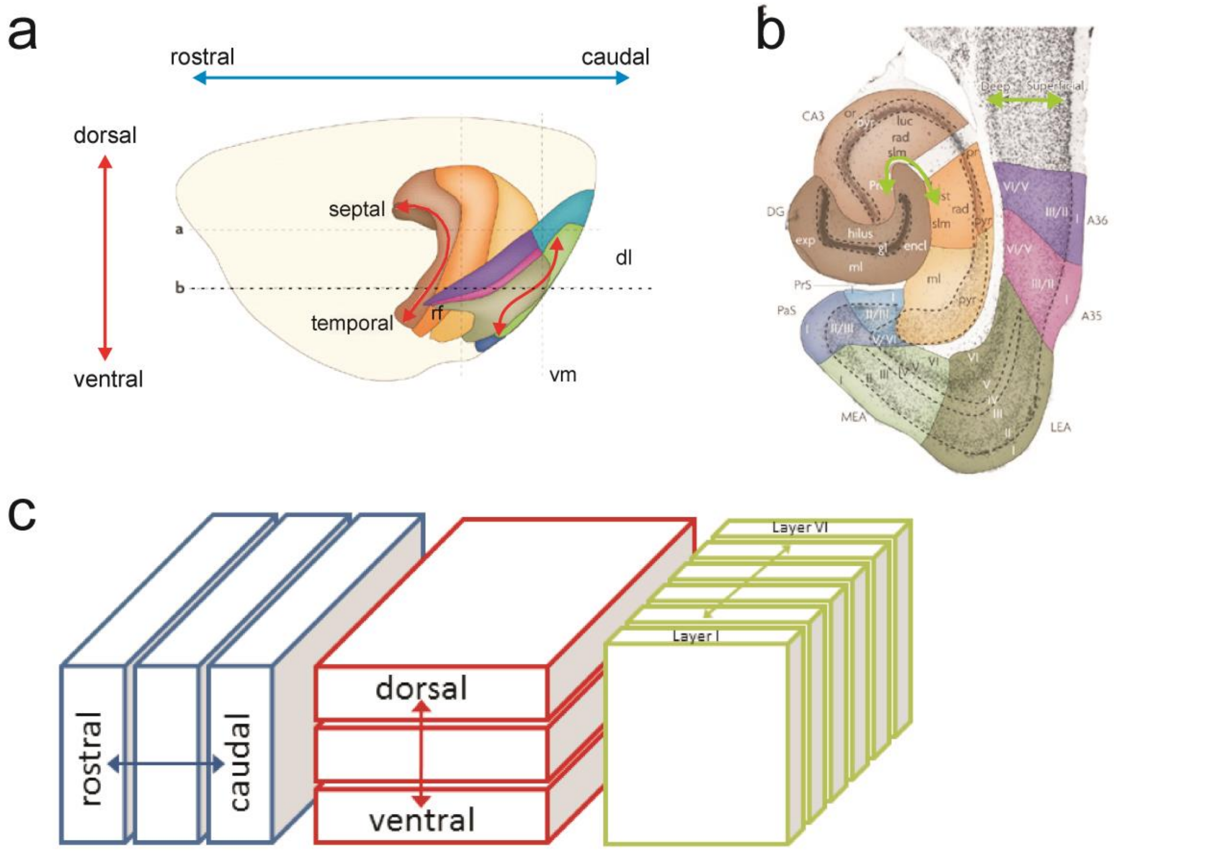

Suppl. Fig. 1

Suppl. Fig. 1 a. representations of the hippocampal formation (HF) and the parahippocampal region (PHR) in the rat brain. A lateral view of the left hemisphere of the rat brain (a). The horizontal dashed line b indicates the level or the horizontal section in b. The following brain regions of the HF are shown: the dentate gyrus (DG; dark brown), CA3 (medium brown), CA1 (orange) and the subiculum (Sub; yellow)). The brain areas of the PHR are: the presubiculum (PrS; medium blue) and parasubiculum (PaS; dark blue), the entorhinal cortex, which has a lateral (LEA; dark green) and a medial (MEA; light green) aspect, the perirhinal cortex (consisting of Brodmann areas (A) 35 (pink) and 36 (purple)) and the postrhinal cortex (POR; blue-green). Three axis are indicated. The first axis, in the dorsoventral dimension is called the dorsoventral axis for PER, POR, the septotemporal axis for the HF areas and dorsolateral-ventromedial axis for the EC. The second axis, in the rostrocaudal dimension is called the transverse or proximodistal axis for the HF areas, PrS and PaS and runs parallel to the cell layer and starts at the DG. For the other areas (EC, PER and POR) this axis is called rostrocaudal axis. The third axis is called the laminar or superficial-to-deep axis, which is defined as being perpendicular to the transverse axis and represents the layers of the HF and PHR areas.

b. A horizontal Nissl-stained section. All subfields of the HF-PHR are color-coded and the axes are indicated as in A. The Roman numerals indicate cortical layers.

b. Displays each of the three spatial positions possible within a subregion. Blue corresponds to the horizontal axis (rostal-caudal), red corresponds to the vertical subdivision in the HF-PHR regions septal-temporal and green depicts the anatomical layers of the HF-PHR regions.

Abbreviations: dist - distal; dl - dorsolateral part of the entorhinal cortex; encl - enclosed blade of the DG; exp - exposed blade of the DG; gl - granule cell layer; luc - stratum lucidum; ml - molecular layer; or - stratum oriens; prox - proximal; pyr - pyramidal cell layer; rad - stratum radiatum; slm - stratum lacunosum-moleculare; vm - ventromedial part of the entorhinal cortex. A and B are adapted from Van Strien, Cappaert & Witter, 2009.

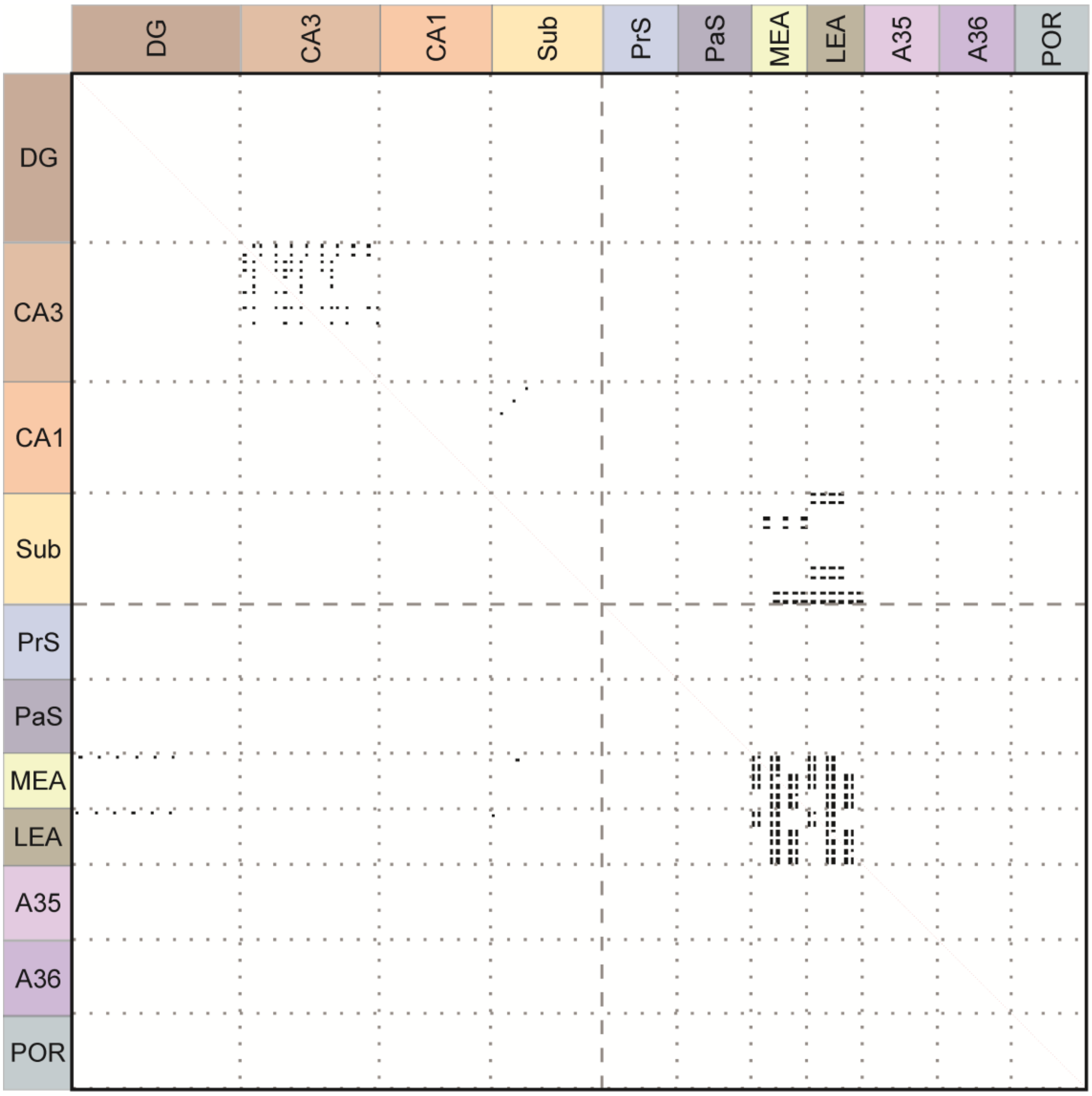

Suppl. Fig. 2

Suppl. Fig. 2: The connections of the hippocampal formation (HF) and parahippocampal region (PHR). A directed binary adjacency matrix comprising 327 nodes in the 11 brain areas of the HF and PHR (color coded). In this case, the brain regions are differentiated within three dimensions and the nodes represent the location specified in three dimensions along the dorsoventral, rostrocaudal and laminar axis. A black dot indicates a connection from an certain area in the row to a particular area in a column.

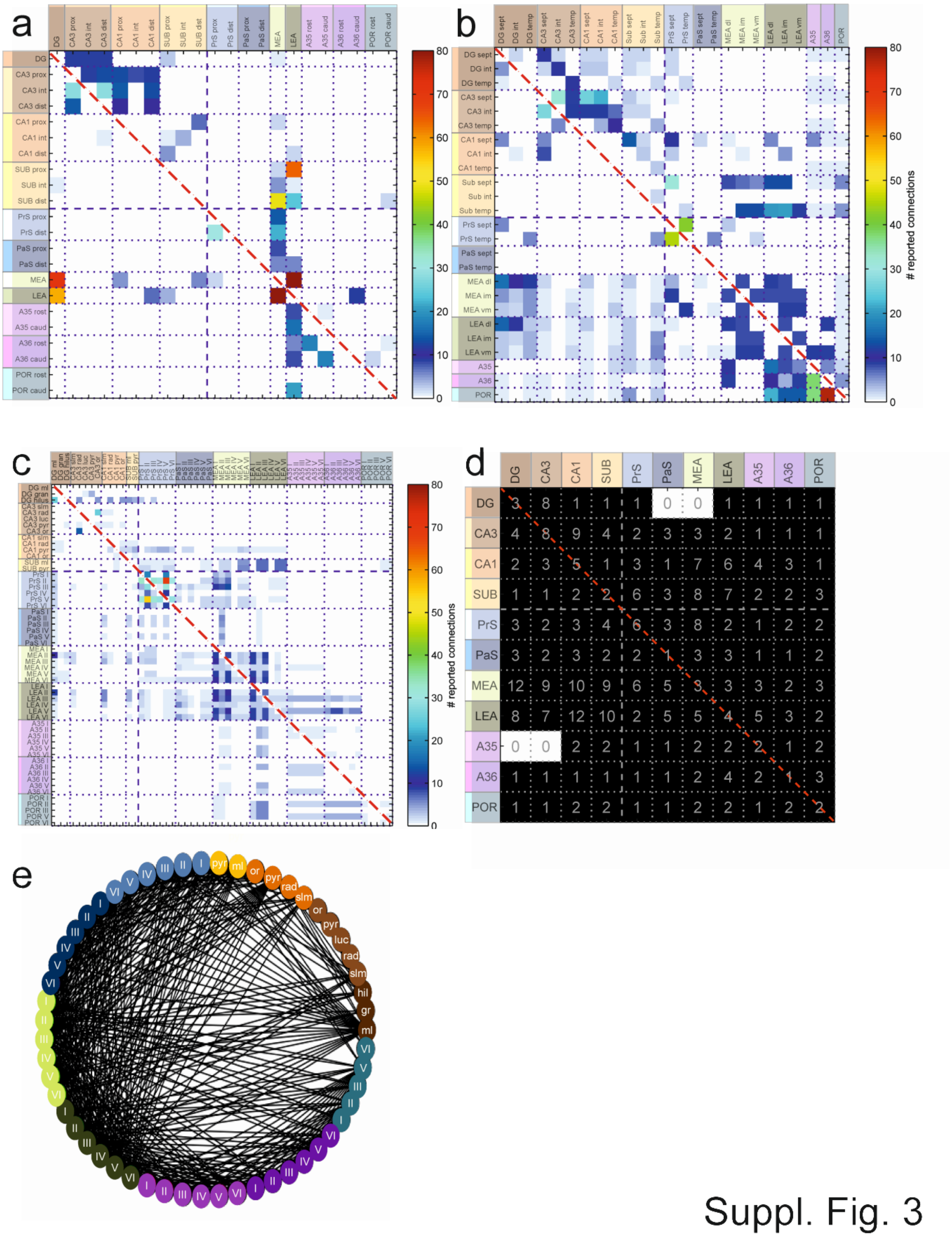

Suppl. Fig. 3: The number of reports in the literature per projection for the connections of the HF-PHR network for the rostrocaudal network (a), the dorsoventral network (b), the laminar network (c). The number of papers reporting on connections between brain areas (d). The laminar network architecture – The connectome Viewer (Gerhard S, Daducci A, Lemkaddem A, Meuli R, Thiran J and Hagmann P (2011). The Connectome Viewer Toolkit: an open source framework to manage, analyze and visualize connectomes. Front. Neuroinform. 5:3. doi: 10.3389/fninf.2011.00003)

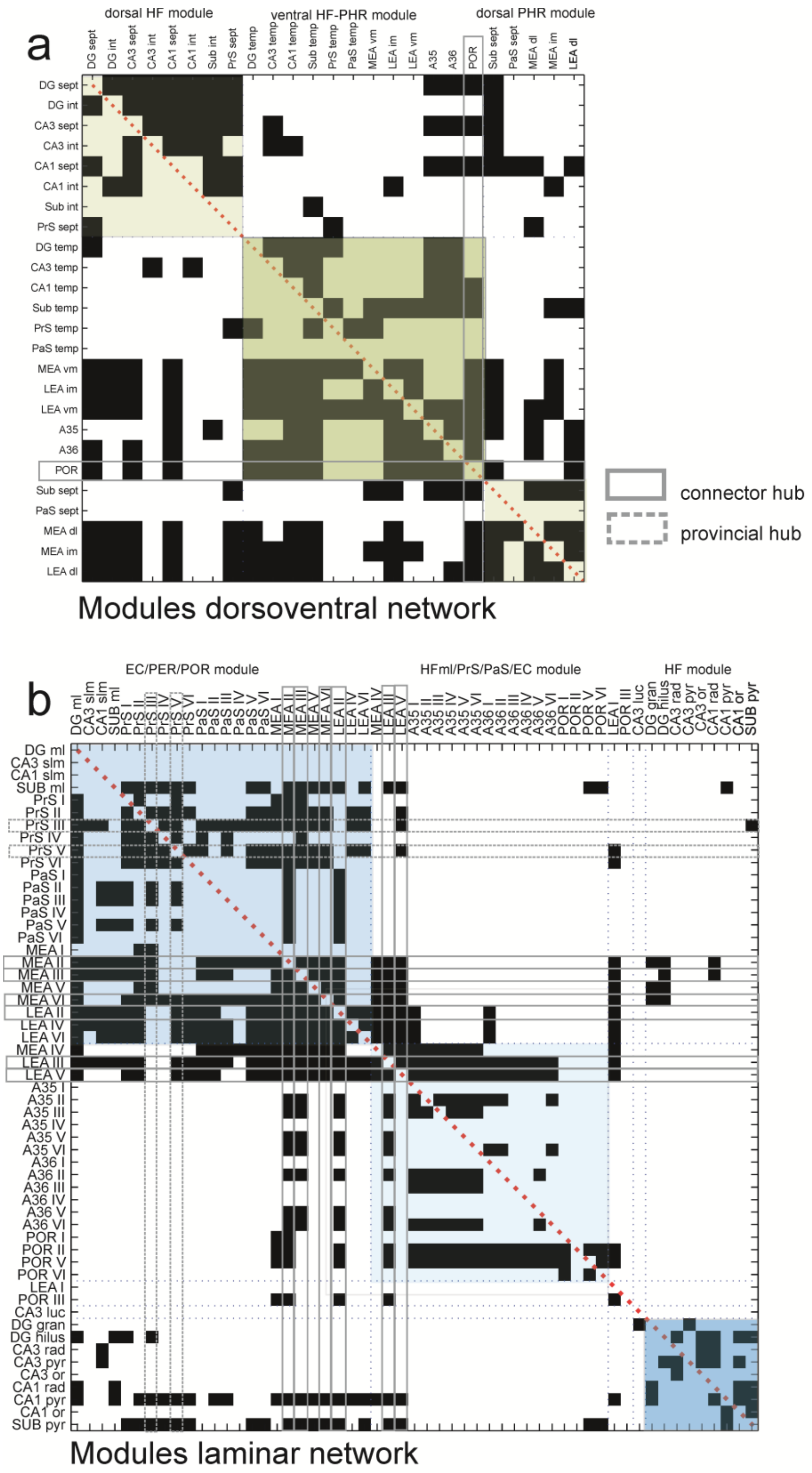

Suppl. Fig. 4

Suppl. Fig. 4: The directed, binary adjacency matrix arranged by the outcome of the modularity analysis of the dorsoventral network (a) and the laminar network (b). The connections of the provincial and connector hubs are indicated with a grey dotted and solid box, respectively.

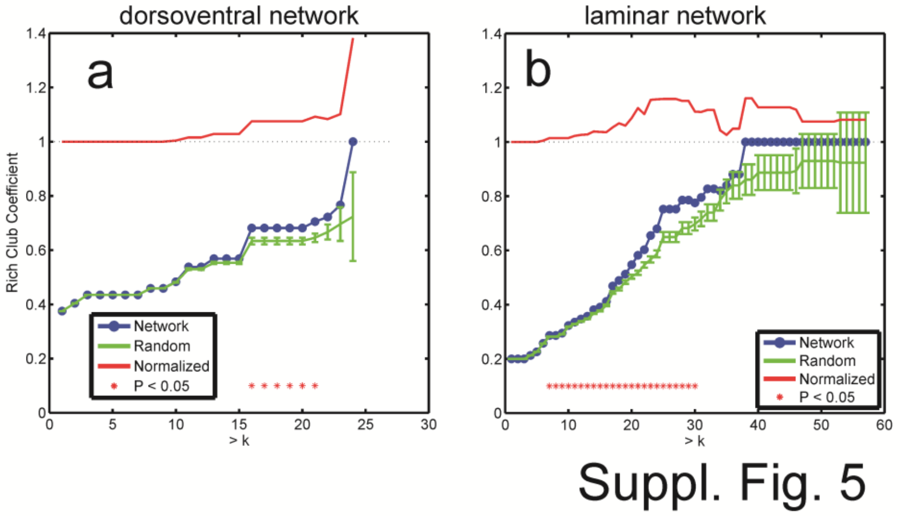

Suppl. Fig. 5: The rich club analysis of the dorsoventral network (a) and the laminar network (b). The rich club coefficients of the real network (blue dots), random network (green lines) and normalized rich club coefficients (red line) are shown. The red stars indicate the *k*'s with a significant rich club.
