# Supplementary material for: Graph analysis of the anatomical network organization of the hippocampal formation and parahippocampal region in the rat
Source: Brain Struct Funct. 2015 Jan 25;221(3):1607–21. doi: 10.1007/s00429-015-0992-0 (PMC4819791; doi:10.1007/s00429-015-0992-0)
Supplement: Supplementary file 2 — Supplementary material 2 (DOCX 46 kb) [file 429_2015_992_MOESM2_ESM.docx]

# Supplementary Material of:

# Graph analysis of the anatomical network organization of the hippocampal formation and parahippocampal region in the rat

F.Z.M. Binicewicz^1^, N.M. van Strien^2^, W.J. Wadman^1^, M.P. van den Heuvel^3,4^, N.L.M. Cappaert^1^

^1^ Swammerdam Institute for Life Science - Center for Neuroscience, University of Amsterdam, Amsterdam, The Netherlands

^2^ Kavli Institute for Systems Neuroscience and Centre for Neural Computation, Norwegian University of Science and Technology, Trondheim, Norway

^3^ Department of Psychiatry, University Medical Center Utrecht, Utrecht, The Netherlands

^4^ Brain Center Rudolf Magnus, University Medical Center Utrecht, Utrecht, The Netherlands

Corresponding author:

N.L.M. Cappaert, PhD

Swammerdam Institute for Life Sciences – Center for Neuroscience

University of Amsterdam

Science Park 904, room C3.266

1098 XH Amsterdam

The Netherlands

email: N.Cappaert@uva.nl

tel.: + 31 20 525 7625

References included in the connectivity database of the hippocampal formation and Parahippocampal region of the rat. See also: [www.templobe-com](http://www.templobe-com)

| **Authors** | **Title** | **Year** | **Journal** | **Vol** | **Pages** | |
| --- | --- | --- | --- | --- | --- | --- |
| Burwell RD;Amaral DG; | Cortical afferents of the perirhinal, postrhinal, and entorhinal cortices of the rat | 1998 | J Comp Neurol | 398 | 179 | 205 |
| Burwell RD;Amaral DG; | Perirhinal and postrhinal cortices of the rat: interconnectivity and connections with the entorhinal cortex | 1998 | J Comp Neurol | 391 | 293 | 321 |
| Kohler C; | A projection from the deep layers of the entorhinal area to the hippocampal formation in the rat brain | 1985 | Neurosci Lett | 56 | 13 | 19 |
| Swanson LW;Kohler C; | Anatomical evidence for direct projections from the entorhinal area to the entire cortical mantle in the rat | 1986 | J Neurosci | 6 | 3010 | 3023 |
| Ruth RE;Collier TJ;Routtenberg A; | Topography between the entorhinal cortex and the dentate septotemporal axis in rats: I. Medial and intermediate entorhinal projecting cells | 1982 | J Comp Neurol | 209 | 69 | 78 |
| Ruth RE;Collier TJ;Routtenberg A; | Topographical relationship between the entorhinal cortex and the septotemporal axis of the dentate gyrus in rats: II. Cells projecting from lateral entorhinal subdivisions | 1988 | J Comp Neurol | 270 | 506 | 516 |
| Steward O;Scoville SA; | Cells of origin of entorhinal cortical afferents to the hippocampus and fascia dentata of the rat | 1976 | J Comp Neurol | 169 | 347 | 370 |
| Nafstad PH; | An electron microscope study on the termination of the perforant path fibres in the hippocampus and the fascia dentata | 1967 | Z Zellforsch Mikrosk Anat | 76 | 532 | 542 |
| Hjorth-Simonsen A;Jeune B; | Origin and termination of the hippocampal perforant path in the rat studied by silver impregnation | 1972 | J Comp Neurol | 144 | 215 | 232 |
| Hjorth-Simonsen A; | Projection of the lateral part of the entorhinal area to the hippocampus and fascia dentata | 1972 | J Comp Neurol | 146 | 219 | 232 |
| Tamamaki N;Nojyo Y; | Projection of the entorhinal layer II neurons in the rat as revealed by intracellular pressure-injection of neurobiotin | 1993 | Hippocampus | 3 | 471 | 480 |
| Naber PA;Witter MP;Lopez da Silva FH; | Perirhinal cortex input to the hippocampus in the rat: evidence for parallel pathways, both direct and indirect. A combined physiological and anatomical study | 1999 | Eur J Neurosci | 11 | 4119 | 4133 |
| Kohler C; | Intrinsic connections of the retrohippocampal region in the rat brain: III. The lateral entorhinal area | 1988 | J Comp Neurol | 271 | 208 | 228 |
| Ishizuka N;Weber J;Amaral DG; | Organization of intrahippocampal projections originating from CA3 pyramidal cells in the rat | 1990 | J Comp Neurol | 295 | 580 | 623 |
| Tamamaki N;Nojyo Y; | Preservation of topography in the connections between the subiculum, field CA1, and the entorhinal cortex in rats | 1995 | J Comp Neurol | 353 | 379 | 390 |
| Tamamaki N; | Organization of the entorhinal projection to the rat dentate gyrus revealed by Dil anterograde labeling | 1997 | Exp Brain Res | 116 | 250 | 258 |
| Amaral DG;Dolorfo C;varez-Royo P; | Organization of CA1 projections to the subiculum: a PHA-L analysis in the rat | 1991 | Hippocampus | 1 | 415 | 435 |
| Witter MP;Holtrop R;van de Loosdrecht AA; | Direct projections from the periallocortical subicular complex to the fascia dentata in the rat. | 1988 | Neurosci Res Comm | 2 | 61 | 68 |
| Kohler C; | Intrinsic projections of the retrohippocampal region in the rat brain. I. The subicular complex | 1985 | J Comp Neurol | 236 | 504 | 522 |
| Kohler C; | Intrinsic connections of the retrohippocampal region in the rat brain. II. The medial entorhinal area | 1986 | J Comp Neurol | 246 | 149 | 169 |
| Caballero-Bleda M;Witter MP; | Regional and laminar organization of projections from the presubiculum and parasubiculum to the entorhinal cortex: an anterograde tracing study in the rat | 1993 | J Comp Neurol | 328 | 115 | 129 |
| Desmond NL;Scott CA;Jane JA;Levy WB; | Ultrastructural identification of entorhinal cortical synapses in CA1 stratum lacunosum-moleculare of the rat | 1994 | Hippocampus | 4 | 594 | 600 |
| Gaarskjaer FB; | Organization of the mossy fiber system of the rat studied in extended hippocampi. I. Terminal area related to number of granule and pyramidal cells | 1978 | J Comp Neurol | 178 | 49 | 72 |
| Gaarskjaer FB; | Organization of the mossy fiber system of the rat studied in extended hippocampi. II. Experimental analysis of fiber distribution with silver impregnation methods | 1978 | J Comp Neurol | 178 | 73 | 88 |
| Deller T;Katona I;Cozzari C;Frotscher M;Freund TF; | Cholinergic innervation of mossy cells in the rat fascia dentata | 1999 | Hippocampus | 9 | 314 | 320 |
| Deller T; | The anatomical organization of the rat fascia dentata: new aspects of laminar organization as revealed by anterograde tracing with Phaseolus vulgaris-Luecoagglutinin (PHAL) | 1998 | Anat Embryol (Berl) | 197 | 89 | 103 |
| Deller T;Adelmann G;Nitsch R;Frotscher M; | The alvear pathway of the rat hippocampus | 1996 | Cell Tissue Res | 286 | 293 | 303 |
| Deller T;Nitsch R;Frotscher M; | Heterogeneity of the commissural projection to the rat dentate gyrus: a Phaseolus vulgaris leucoagglutinin tracing study | 1996 | Neuroscience | 75 | 111 | 121 |
| Caballero-Bleda M;Witter MP; | Projections from the presubiculum and the parasubiculum to morphologically characterized entorhinal-hippocampal projection neurons in the rat | 1994 | Exp Brain Res | 101 | 93 | 108 |
| Claiborne BJ;Amaral DG;Cowan WM; | A light and electron microscopic analysis of the mossy fibers of the rat dentate gyrus | 1986 | J Comp Neurol | 246 | 435 | 458 |
| Deller T;Nitsch R;Frotscher M; | Layer-specific sprouting of commissural fibres to the rat fascia dentata after unilateral entorhinal cortex lesion: a Phaseolus vulgaris leucoagglutinin tracing study | 1996 | Neuroscience | 71 | 651 | 660 |
| Swanson LW;Wyss JM;Cowan WM; | An autoradiographic study of the organization of intrahippocampal association pathways in the rat | 1978 | J Comp Neurol | 181 | 681 | 715 |
| Deller T;Frotscher M;Nitsch R; | Sprouting of crossed entorhinodentate fibers after a unilateral entorhinal lesion: anterograde tracing of fiber reorganization with Phaseolus vulgaris-leucoagglutinin (PHAL) | 1996 | J Comp Neurol | 365 | 42 | 55 |
| van Groen T;Wyss JM; | Extrinsic projections from area CA1 of the rat hippocampus: olfactory, cortical, subcortical, and bilateral hippocampal formation projections | 1990 | J Comp Neurol | 302 | 515 | 528 |
| Deller T;Frotscher M;Nitsch R; | Morphological evidence for the sprouting of inhibitory commissural fibers in response to the lesion of the excitatory entorhinal input to the rat dentate gyrus | 1995 | J Neurosci | 15 | 6868 | 6878 |
| Deller T;Nitsch R;Frotscher M; | Phaseolus vulgaris-leucoagglutinin tracing of commissural fibers to the rat dentate gyrus: evidence for a previously unknown commissural projection to the outer molecular layer | 1995 | J Comp Neurol | 352 | 55 | 68 |
| Steward O; | Topographic organization of the projections from the entorhinal area to the hippocampal formation of the rat | 1976 | J Comp Neurol | 167 | 285 | 314 |
| Finch DM;Babb TL; | Demonstration of caudally directed hippocampal efferents in the rat by intracellular injection of horseradish peroxidase | 1981 | Brain Res | 214 | 405 | 410 |
| Finch DM;Nowlin NL;Babb TL; | Demonstration of axonal projections of neurons in the rat hippocampus and subiculum by intracellular injection of HRP | 1983 | Brain Res | 271 | 201 | 216 |
| Harris E;Witter MP;Weinstein G;Stewart M; | Intrinsic connectivity of the rat subiculum: I. Dendritic morphology and patterns of axonal arborization by pyramidal neurons | 2001 | J Comp Neurol | 435 | 490 | 505 |
| Honda Y;Ishizuka N; | Organization of connectivity of the rat presubiculum: I. Efferent projections to the medial entorhinal cortex | 2004 | J Comp Neurol | 473 | 463 | 484 |
| Naber PA;Caballero-Bleda M;Jorritsma-Byham B;Witter MP; | Parallel input to the hippocampal memory system through peri- and postrhinal cortices | 1997 | Neuroreport | 8 | 2617 | 2621 |
| Swanson LW;Cowan WM; | An autoradiographic study of the organization of the efferent connections of the hippocampal formation in the rat | 1977 | J Comp Neurol | 172 | 49 | 84 |
| Tamamaki N;Watanabe K;Nojyo Y; | A whole image of the hippocampal pyramidal neuron revealed by intracellular pressure-injection of horseradish peroxidase | 1984 | Brain Res | 307 | 336 | 340 |
| Tamamaki N;Abe K;Nojyo Y; | Columnar organization in the subiculum formed by axon branches originating from single CA1 pyramidal neurons in the rat hippocampus | 1987 | Brain Res | 412 | 156 | 160 |
| van Groen T;Wyss JM; | The postsubicular cortex in the rat: characterization of the fourth region of the subicular cortex and its connections | 1990 | Brain Res | 529 | 165 | 177 |
| van Groen T;Wyss JM; | The connections of presubiculum and parasubiculum in the rat | 1990 | Brain Res | 518 | 227 | 243 |
| Segal M;Landis S; | Afferents to the hippocampus of the rat studied with the method of retrograde transport of horseradish peroxidase | 1974 | Brain Res | 78 | 1 | 15 |
| Naber PA;Lopes da Silva FH;Witter MP; | Reciprocal connections between the entorhinal cortex and hippocampal fields CA1 and the subiculum are in register with the projections from CA1 to the subiculum | 2001 | Hippocampus | 11 | 99 | 104 |
| Kosel KC;Van Hoesen GW;Rosene DL; | A direct projection from the perirhinal cortex (area 35) to the subiculum in the rat | 1983 | Brain Res | 269 | 347 | 351 |
| Insausti R;Herrero MT;Witter MP; | Entorhinal cortex of the rat: cytoarchitectonic subdivisions and the origin and distribution of cortical efferents | 1997 | Hippocampus | 7 | 146 | 183 |
| Van Haeften T;Wouterlood FG;Jorritsma-Byham B;Witter MP; | GABAergic presubicular projections to the medial entorhinal cortex of the rat | 1997 | J Neurosci | 17 | 862 | 874 |
| Van Haeften T;Wouterlood FG;Witter MP; | Presubicular input to the dendrites of layer-V entorhinal neurons in the rat | 2000 | Ann N Y Acad Sci | 911 | 471 | 473 |
| Dolorfo CL;Amaral DG; | Entorhinal cortex of the rat: organization of intrinsic connections | 1998 | J Comp Neurol | 398 | 49 | 82 |
| Dolorfo CL;Amaral DG; | Entorhinal cortex of the rat: topographic organization of the cells of origin of the perforant path projection to the dentate gyrus | 1998 | J Comp Neurol | 398 | 25 | 48 |
| Tamamaki N;Abe K;Nojyo Y; | Three-dimensional analysis of the whole axonal arbors originating from single CA2 pyramidal neurons in the rat hippocampus with the aid of a computer graphic technique | 1988 | Brain Res | 452 | 255 | 272 |
| Li XG;Somogyi P;Ylinen A;Buzsaki G; | The hippocampal CA3 network: an in vivo intracellular labeling study | 1994 | J Comp Neurol | 339 | 181 | 208 |
| Matsuda S;Kobayashi Y;Ishizuka N; | A quantitative analysis of the laminar distribution of synaptic boutons in field CA3 of the rat hippocampus | 2004 | Neurosci Res | 49 | 241 | 252 |
| Hjorth-Simonsen A; | Hippocampal efferents to the ipsilateral entorhinal area: an experimental study in the rat | 1971 | J Comp Neurol | 142 | 417 | 437 |
| Cenquizca LA;Swanson LW; | Spatial organization of direct hippocampal field CA1 axonal projections to the rest of the cerebral cortex | 2007 | Brain Res Rev | 56 | 1 | 26 |
| Sik A;Tamamaki N;Freund TF; | Complete axon arborization of a single CA3 pyramidal cell in the rat hippocampus, and its relationship with postsynaptic parvalbumin-containing interneurons | 1993 | Eur J Neurosci | 5 | 1719 | 1728 |
| Siddiqui AH;Joseph SA; | CA3 axonal sprouting in kainate-induced chronic epilepsy | 2005 | Brain Res | 1066 | 129 | 146 |
| Hargreaves EL;Rao G;Lee I;Knierim JJ; | Major dissociation between medial and lateral entorhinal input to dorsal hippocampus | 2005 | Science | 308 | 1792 | 1794 |
| Kerr KM;Agster KL;Furtak SC;Burwell RD; | Functional neuroanatomy of the parahippocampal region: The lateral and medial entorhinal areas | 2007 | Hippocampus | 17 | 697 | 708 |
| Furtak SC;Wei SM;Agster KL;Burwell RD; | Functional neuroanatomy of the parahippocampal region in the rat: The perirhinal and postrhinal cortices | 2007 | Hippocampus | 17 | 709 | 722 |
| Laurberg S;Sorensen KE; | Associational and commissural collaterals of neurons in the hippocampal formation (hilus fasciae dentatae and subfield CA3) | 1981 | Brain Res | 212 | 287 | 300 |
| Laurberg S; | Commissural and intrinsic connections of the rat hippocampus | 1979 | J Comp Neurol | 184 | 685 | 708 |
| Blackstad TW;Brink K;Hem J;Jeune B; | Distribution of hippocampal mossy fibers in the rat. An experimental study with silver impregnation methods | 1970 | J Comp Neurol | 138 | 433 | 449 |
| Ishizuka N; | Laminar organization of the pyramidal cell layer of the subiculum in the rat | 2001 | J Comp Neurol | 435 | 89 | 110 |
| Deacon TW;Eichenbaum H;Rosenberg P;Eckmann KW; | Afferent connections of the perirhinal cortex in the rat | 1983 | J Comp Neurol | 220 | 168 | 190 |
| Wittner L;Henze DA;Zaborszky L;Buzsaki G; | Hippocampal CA3 pyramidal cells selectively innervate aspiny interneurons | 2006 | Eur J Neurosci | 24 | 1286 | 1298 |
| Segal M; | Afferents to the entorhinal cortex of the rat studied by the method of retrograde transport of horseradish peroxidase | 1977 | Exp Neurol | 57 | 750 | 765 |
| Soriano E;Frotscher M; | A GABAergic axo-axonic cell in the fascia dentata controls the main excitatory hippocampal pathway | 1989 | Brain Res | 503 | 170 | 174 |
| Beckstead RM; | Afferent connections of the entorhinal area in the rat as demonstrated by retrograde cell-labeling with horseradish peroxidase | 1978 | Brain Res | 152 | 249 | 264 |
| Baks-Te-Bulte L;Wouterlood FG;Vinkenoog M;Witter MP; | Entorhinal projections terminate onto principal neurons and interneurons in the subiculum: a quantitative electron microscopical analysis in the rat | 2005 | Neuroscience | 136 | 729 | 739 |
| Naber PA;Witter MP; | Subicular efferents are organized mostly as parallel projections: a double-labeling, retrograde-tracing study in the rat | 1998 | J Comp Neurol | 393 | 284 | 297 |
| Wyss JM; | An autoradiographic study of the efferent connections of the entorhinal cortex in the rat | 1981 | J Comp Neurol | 199 | 495 | 512 |
| Honda Y;Umitsu Y;Ishizuka N; | Efferent projections of the subiculum to the retrohippocampal and retrosplenial cortices of the rat | 1999 | Neurosci Res | S265 |  |  |
| Honda Y;Ishizuka N; | The organization of the intrinsic and entorhinal connections of the rat presubiculum | 1998 | Neurosci Res | S275 |  |  |
| Honda Y;Umitsu Y;Ishizuka N; | Topographic projections of perforant path from entorhinal area to CA1 and subiculum in the rat | 2000 | Neurosci Res | S101 |  |  |
| Kajiwara R;Wouterlood FG;Sah A;Boekel AJ;Baks-Te Bulte LT;Witter MP; | Convergence of entorhinal and CA3 inputs onto pyramidal neurons and interneurons in hippocampal area CA1-An anatomical study in the rat | 2008 | Hippocampus | 18 | 266 | 280 |
| Kloosterman F;Witter MP;Van HT; | Topographical and laminar organization of subicular projections to the parahippocampal region of the rat | 2003 | J Comp Neurol | 455 | 156 | 171 |
| Frotscher M;Seress L;Schwerdtfeger WK;Buhl E; | The mossy cells of the fascia dentata: a comparative study of their fine structure and synaptic connections in rodents and primates | 1991 | J Comp Neurol | 312 | 145 | 163 |
| Lingenhohl K;Finch DM; | Morphological characterization of rat entorhinal neurons in vivo: soma-dendritic structure and axonal domains | 1991 | Exp Brain Res | 84 | 57 | 74 |
| Naber PA;Witter MP;Lopes da Silva FH; | Evidence for a direct projection from the postrhinal cortex to the subiculum in the rat | 2001 | Hippocampus | 11 | 105 | 117 |
| Lubke J;Deller T;Frotscher M; | Septal innervation of mossy cells in the hilus of the rat dentate gyrus: an anterograde tracing and intracellular labeling study | 1997 | Exp Brain Res | 114 | 423 | 432 |
| Honda Y;Umitsu Y;Ishizuka N; | Organization of connectivity of the rat presubiculum: II. Associational and commissural connections | 2008 | J Comp Neurol | 506 | 640 | 658 |
| Wouterlood FG;Van HT;Eijkhoudt M;Baks-Te-Bulte L;Goede PH;Witter MP; | Input from the presubiculum to dendrites of layer-V neurons of the medial entorhinal cortex of the rat | 2004 | Brain Res | 1013 | 1 | 12 |
| Van Haeften T;Baks-Te-Bulte L;Goede PH;Wouterlood FG;Witter MP; | Morphological and numerical analysis of synaptic interactions between neurons in deep and superficial layers of the entorhinal cortex of the rat | 2003 | Hippocampus | 13 | 943 | 952 |
| Witter MP;Ostendorf RH;Groenewegen HJ; | Heterogeneity in the Dorsal Subiculum of the Rat. Distinct Neuronal Zones Project to Different Cortical and Subcortical Targets | 1990 | Eur J Neurosci | 2 | 718 | 725 |
| Witter MP;Groenewegen HJ; | The subiculum: cytoarchitectonically a simple structure, but hodologically complex | 1990 | Prog Brain Res | 83 | 47 | 58 |
| Van Haeften T;Jorritsma-Byham B;Witter MP; | Quantitative morphological analysis of subicular terminals in the rat entorhinal cortex | 1995 | Hippocampus | 5 | 452 | 459 |
| Swanson LW;Sawchenko PE;Cowan WM; | Evidence for collateral projections by neurons in Ammon's horn, the dentate gyrus, and the subiculum: a multiple retrograde labeling study in the rat | 1981 | J Neurosci | 1 | 548 | 559 |
| Sorra KE;Harris KM; | Occurrence and three-dimensional structure of multiple synapses between individual radiatum axons and their target pyramidal cells in hippocampal area CA1 | 1993 | J Neurosci | 13 | 3736 | 3748 |
| Shi CJ;Cassell MD; | Perirhinal cortex projections to the amygdaloid complex and hippocampal formation in the rat | 1999 | J Comp Neurol | 406 | 299 | 328 |
| McIntyre DC;Kelly ME;Staines WA; | Efferent projections of the anterior perirhinal cortex in the rat | 1996 | J Comp Neurol | 369 | 302 | 318 |
| Deller T;Leranth C; | Synaptic connections of neuropeptide Y (NPY) immunoreactive neurons in the hilar area of the rat hippocampus | 1990 | J Comp Neurol | 300 | 433 | 447 |
| Deller T;Nitsch R;Frotscher M; | Associational and commissural afferents of parvalbumin-immunoreactive neurons in the rat hippocampus: a combined immunocytochemical and PHA-L study | 1994 | J Comp Neurol | 350 | 612 | 622 |
| Deller T;Martinez A;Nitsch R;Frotscher M; | A novel entorhinal projection to the rat dentate gyrus: direct innervation of proximal dendrites and cell bodies of granule cells and GABAergic neurons | 1996 | J Neurosci | 16 | 3322 | 3333 |
| Deller T;Del TD;Rappert A;Bechmann I; | Structural reorganization of the dentate gyrus following entorhinal denervation: species differences between rat and mouse | 2007 | Prog Brain Res | 163 | 501 | 528 |
| Eid T;Jorritsma-Byham B;Schwarcz R;Witter MP; | Afferents to the seizure-sensitive neurons in layer III of the medial entorhinal area: a tracing study in the rat | 1996 | Exp Brain Res | 109 | 209 | 218 |
| Hjorth-Simonsen A; | Some intrinsic connections of the hippocampus in the rat: an experimental analysis | 1973 | J Comp Neurol | 147 | 145 | 161 |
| Kohler C; | Morphological details of the projection from the presubiculum to the entorhinal area as shown with the novel PHA-L immunohistochemical tracing method in the rat | 1984 | Neurosci Lett | 45 | 285 | 290 |
| Beggs JM;Moyer JR;McGann JP;Brown TH; | Prolonged synaptic integration in perirhinal cortical neurons | 2000 | J Neurophysiol | 83 | 3294 | 3298 |
| White TD;Tan AM;Finch DM; | Functional reciprocal connections of the rat entorhinal cortex and subicular complex with the medial frontal cortex: an in vivo intracellular study | 1990 | Brain Res | 533 | 95 | 106 |
| Groenewegen HJ;Vermeulen-Van der ZE;te KA;Witter MP; | Organization of the projections from the subiculum to the ventral striatum in the rat. A study using anterograde transport of Phaseolus vulgaris leucoagglutinin | 1987 | Neuroscience | 23 | 103 | 120 |
| Meibach RC;Siegel A; | Efferent connections of the hippocampal formation in the rat | 1977 | Brain Res | 124 | 197 | 224 |
| Meibach RC;Siegel A; | Subicular projections to the posterior cingulate cortex in rats | 1977 | Exp Neurol | 57 | 264 | 274 |
| Tamamaki N;Nojyo Y; | Disposition of the slab-like modules formed by axon branches originating from single CA1 pyramidal neurons in the rat hippocampus | 1990 | J Comp Neurol | 291 | 509 | 519 |
| Andersen P;Bliss TV;Lomo T;Olsen LI;Skrede KK; | Lamellar organization of hippocampal excitatory pathways | 1969 | Acta Physiol Scand | 76 | 4A | 5A |
| Gottlieb DI;Cowan WM; | Autoradiographic studies of the commissural and ipsilateral association connection of the hippocampus and detentate gyrus of the rat. I. The commissural connections | 1973 | J Comp Neurol | 149 | 393 | 421 |
| Raisman G;Cowan WM;Powell TP; | An experimental analysis of the efferent projection of the hippocampus | 1966 | Brain | 89 | 83 | 108 |
| Van GT;Wyss JM; | Species differences in hippocampal commissural connections: studies in rat, guinea pig, rabbit, and cat | 1988 | J Comp Neurol | 267 | 322 | 334 |
| Wittner L;Henze DA;Zaborszky L;Buzsaki G; | Three-dimensional reconstruction of the axon arbor of a CA3 pyramidal cell recorded and filled in vivo | 2007 | Brain Struct Funct | 212 | 75 | 83 |
| Swanson LW;Sawchenko PE;Cowan WM; | Evidence that the commissural, associational and septal projections of the regio inferior of the hippocampus arise from the same neurons | 1980 | Brain Res | 197 | 207 | 212 |
| Witter MP;Griffioen AW;Jorritsma-Byham B;Krijnen JL; | Entorhinal projections to the hippocampal CA1 region in the rat: an underestimated pathway | 1988 | Neurosci Lett | 85 | 193 | 198 |
| Kohler C;Shipley MT;Srebro B;Harkmark W; | Some retrohippocampal afferents to the entorhinal cortex. Cells of origin as studied by the HRP method in the rat and mouse | 1978 | Neurosci Lett | 10 | 115 | 120 |
